# Supplementary material for: Synergistic Lanthanum-Cysteine Chelate and Corn Steep Liquor Mitigate Cadmium Toxicity in Chinese Cabbage via Physiological–Microbial Coordination
Source: Plants (Basel). 2025 Oct 1;14(19):3040. doi: 10.3390/plants14193040 (PMC12525533; doi:10.3390/plants14193040)
Supplement: Supplementary file 1 [file plants-14-03040-s001.zip › plants-3848781-supplementary.pdf]

Synergistic Lanthanum-Cysteine Chelate and Corn Steep Liquor Mitigate Cadmium Toxicity in Chinese Cabbage via Physiological-Microbial Coordination

Fengbo Ma [1, †], Zihao Wang [1, †], Wenhao Wang [1], Xian Wang [1], Xiaojing Ma [1], Xinjun Zhang [1], Yanli Liu [1], Qing Chen [1], Kangguo Mu [1,*]

[1] *Beijing Key Laboratory of Farmyard Soil Pollution Prevention-Control and Remediation, College of Resources and Environmental Sciences, China Agricultural University, Beijing 100193, China.*

[†] These authors contributed to the work equally and should be regarded as co-first authors.

* Corresponding author.

Email address: kgmu@cau.edu.cn

Figure captions:

Figure S1 Plant growth parameters of different treatments

plant height (a), root length (b), fresh weight (c), dry weight (d). CLa0.1 significantly increased plant height (10.9%) and CLa0.5 promoted root length (21.5%), while CLa2.5-12.5 notably improved biomass accumulation.

Figure S2 Photosynthetic pigment content of different treatments

chlorophyll a (a), chlorophyll b (b), carotenoids (c). CLa2.5 markedly enhanced chlorophyll a and carotenoids by 21.1% and 18.2%, respectively, indicating improved photosynthetic performance under Cd stress.

Figure S3 Cd concentration in soil and plants

DTPA-Cd concentration in soil (a), shoot Cd concentration (b), root Cd concentration (c). CLa2.5 significantly decreased soil available Cd (by 5.7%) and reduced shoot and root Cd concentrations by 26.8% and 27.2%, respectively.

Figure S4 Alpha diversity indices plot of rhizosphere microbial communities in Chinese cabbage under different treatments.

Richness (a), Shannon (b), Chao (c)

2. Materials and Methods

2.3.1. Plant Growth Parameters

The vegetative growth parameters were quantified as follows: plant height was determined as the vertical distance from the crown base to the apex of the longest leaf, root length was measured from the root crown to the terminal tip of the primary root system, aboveground fresh weight was recorded immediately after harvest, aboveground dry weight was obtained through sequential desiccation at 105 °C for 30 min followed by 65 °C for 48 h prior to gravimetric analysis.

Experimental Setup

Cd-contaminated soil was collected from agricultural fields in Xiangtan City, Hunan Province (27°94′41″N, 112°97′08″E). Visible impurities (e.g., gravel, plant residues) were manually removed prior to analysis. The soil exhibited the following characteristics: pH 5.31, EC 292 μS cm^{-1}, organic matter (OM) 41.6 g kg^{-1}, alkaline nitrogen (AN) 129 mg kg^{-1}, available phosphorus (AP) 29.7 mg kg^{-1}, exchangeable potassium (EK) 219 mg kg^{-1}, total Cd 2.15 mg kg^{-1}, and bioavailable Cd 1.51 mg kg^{-1}.

Chinese cabbage (*Brassica rapa subsp. pekinensis* Cv. 'Jing Cui 60') was cultivated in a controlled greenhouse under the following conditions: 16/8 h light/dark photoperiod, 70-75% relative humidity, 700 ± 20 μmol m^{-2} s^{-1} photosynthetically active radiation (PAR), and 26/20 °C day/night temperatures. The experimental design included seven treatments: (i) Control (CK, deionized water), (ii) CLa0.1, (iii) CLa0.5, (iv) CLa2.5, (v) CLa12.5, (vi) CLa25, (vii) CLa62.5 (Table. S1). Each treatment was replicated three times in a completely randomized design. Surface-sterilized seeds were sown in commercial substrate (Pindstrup, Denmark, pH 5.5–6.6, OM 177 g kg^{-1}, CEC 26.9 mol kg^{-1}, inorganic N 29.9 mg kg^{-1}, P 190 mg kg^{-1}, and K 9.42 mg kg^{-1}). Seedings at the two-true-leaf stage were transplanted into pots (14 cm top diameter × 10 cm bottom diameter × 16 cm height) containing 800 g of soil. Soil moisture was maintained at 65% of water-holding capacity through daily irrigation. Starting 7 days after transplantation, 100 mL of treatment solution was applied to the root zone at 7-day intervals for three consecutive weeks. A 30-10-10 NPK compound fertilizer (0.5 g kg^{-1} soil) was applied on day 15. After 30 days of growth, plants were harvested and separated into shoots and roots, and rhizosphere soil samples were collected and stored at -80 °C for subsequent analysis.

Table S1. Experimental treatments

Treatment	CLa (mg kg^{-1})

CK	0
CLa0.1	0.1
CLa0.5	0.5
CLa2.5	2.5
CLa12.5	12.5
CLa25	25
CLa62.6	62.5

3. Results

In our preliminary study, different CLa concentrations were tested to determine the optimal application level under Cd stress. CLa showed a dose-dependent effect on Chinese cabbage growth, with promotion at 0.1-12.5 mg kg^{-1}, but no significant effect at 25 and 62.5 mg kg^{-1}. The most pronounced increase in plant height (10.9% over control) occurred at 0.1 mg kg^{-1} CLa (Figure S1a), while root length was maximally enhanced at 0.5 mg kg^{-1} (21.5% increase, Figure S1b). Fresh weight was significantly improved across all treatments within 0.1-12.5 mg kg^{-1} (7.3–8.9% increase), and at 12.5 mg kg^{-1}, dry weight also increased by 9.4% compared to control. In terms of photosynthetic pigments, 2.5 mg·kg^{-1} CLa showed the most pronounced effect, significantly increasing chlorophyll a and carotenoid contents by 21.1% and 18.2%, respectively. Moreover, it was most effective in promoting soil Cd immobilization and reducing Cd uptake in Chinese cabbage, as evidenced by a 5.7% decrease in soil DTPA-Cd concentration and significant reductions of 26.8% and 27.2% in root and shoot Cd concentrations, respectively.

In conclusion, CLa2.5 was chosen for subsequent experiments, as it exhibited the most effective overall performance in alleviating Cd stress in Chinese cabbage.

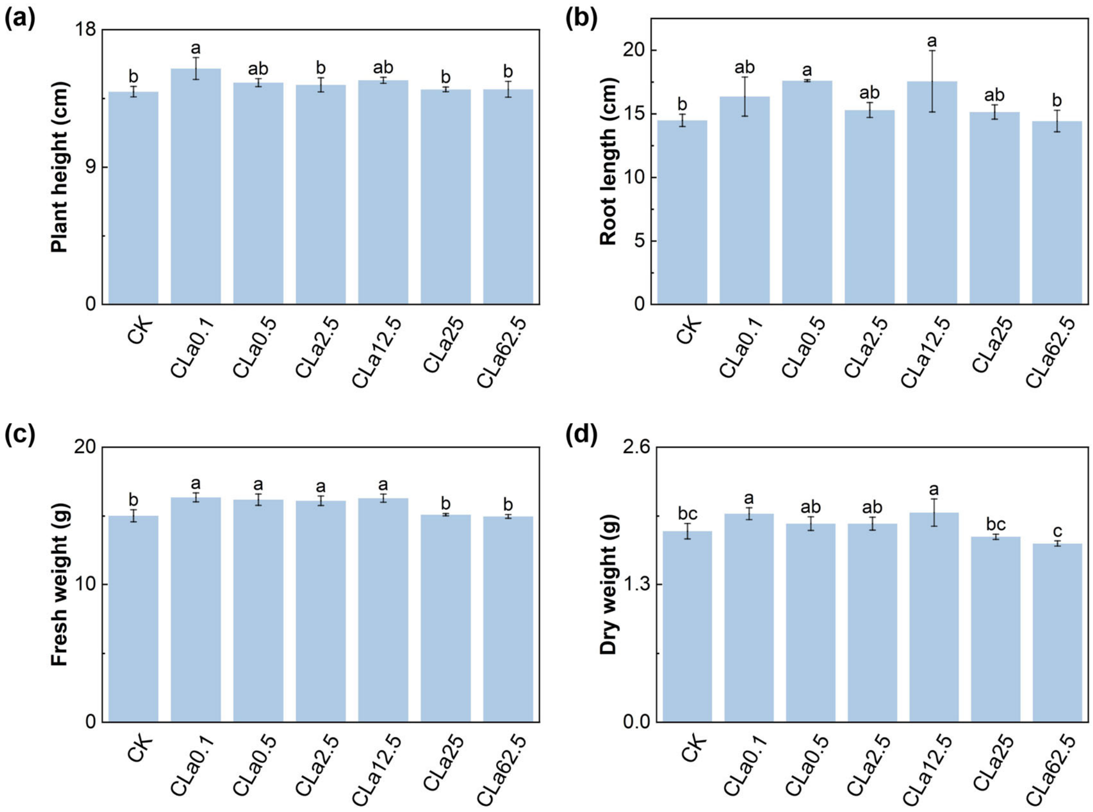

Figure S1 Effect of different treatments (CK: deionized water, CLa0.1: 0.1 mg kg^{-1} CLa, CLa0.5: 0.5 mg kg^{-1} CLa, CLa2.5: 2.5 mg kg^{-1} CLa, CLa12.5: 12.5 mg kg^{-1} CLa, CLa25: 25 mg kg^{-1} CLa, CLa62.5: 62.5 mg kg^{-1} CLa) on plant height (a), root length (b), fresh weight (c), dry weight (d) Different lowercase letters indicate significant differences among treatments ($P < 0.05$; n = 3)

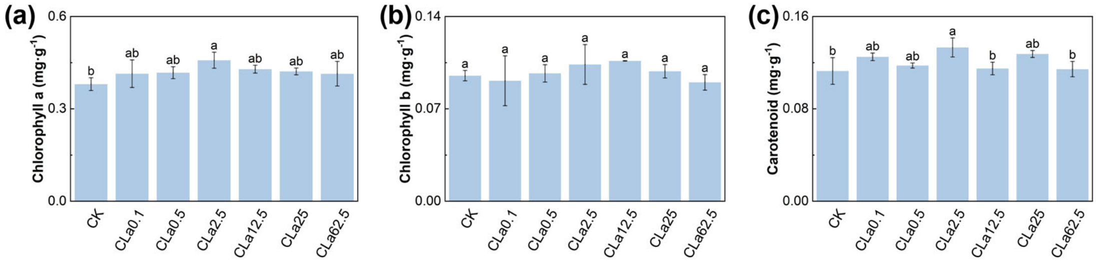

Figure S2 Effect of different treatments (CK: deionized water, CLa0.1: 0.1 mg kg^{-1} CLa, CLa0.5: 0.5 mg kg^{-1} CLa, CLa2.5: 2.5 mg kg^{-1} CLa, CLa12.5: 12.5 mg kg^{-1} CLa, CLa25: 25 mg kg^{-1} CLa, CLa62.5: 62.5 mg kg^{-1} CLa) on chlorophyll a (a), chlorophyll b (b), carotenoids (c) Different lowercase letters indicate significant differences among treatments ($P < 0.05$; n = 3)

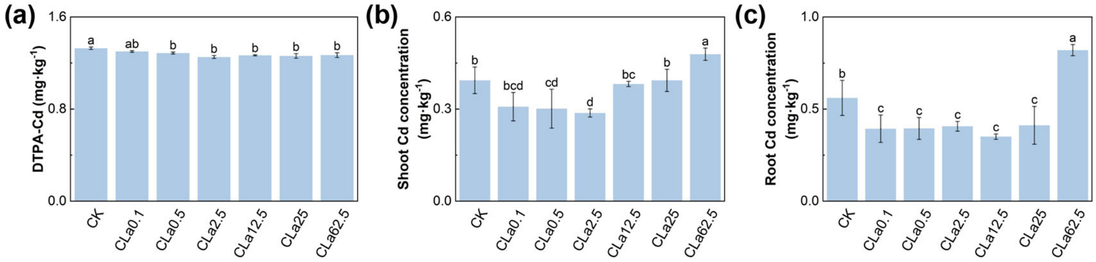

Figure S3 Effect of different treatments (CK: deionized water, CLa0.1: 0.1 mg kg^{-1} CLa, CLa0.5: 0.5 mg kg^{-1} CLa, CLa2.5: 2.5 mg kg^{-1} CLa, CLa12.5: 12.5 mg kg^{-1} CLa, CLa25: 25 mg kg^{-1} CLa, CLa62.5: 62.5 mg kg^{-1} CLa) on DTPA-Cd concentration in soil (a), shoot Cd concentration (b), root Cd concentration (c). Different lowercase letters indicate significant differences among treatments ($P < 0.05$; n = 3)

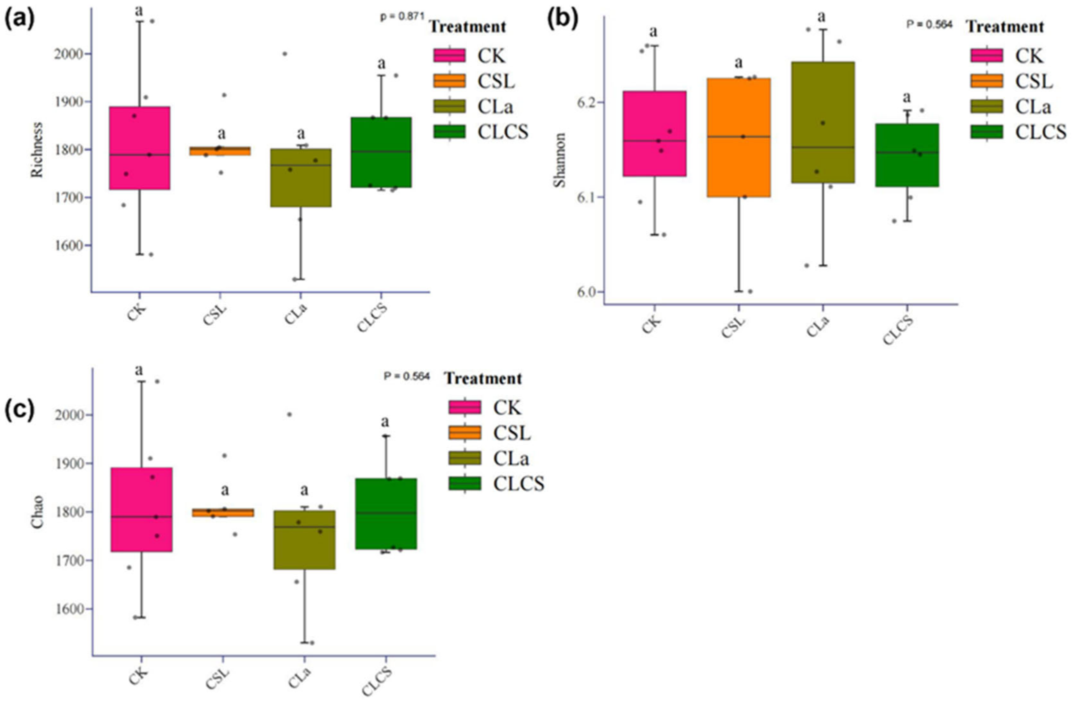

Figure S4 The alpha diversity of the bacterial community in soil. Richness (a), Shannon (b), Chao (c). Different lowercase letters indicate significant differences among treatments ($P < 0.05$; n = 6).

Table S2. The alpha diversity of the bacterial community in soil

Treatment	Richness	Chao1 Index	Shannon Index
CK	1838 a	1859 a	8.90 a
CLa	1754 a	1785 a	8.88 a
CSL	1773 a	1811 a	8.85 a
CLCS	1808 a	1857 a	8.85 a

Different lowercase letters indicate significant differences among treatments ($P < 0.05$; n = 6).

Table S3 Results of ANOVA (MS, F value) for each parameter

	ANOVA	
Figure	MS	F
Plant Height	0.690	2.090
Root Length	8.336	3.128
Fresh Weight	117.014	17.226
Dry Weight	0.274	2.098
Surface root area	6852.891	10.471
Root volume	1.238	6.693
Root tips	1815295.417	5.241
Root forks	46470343.19	4.533
Root crossing	4067049.861	4.008
Root activity	6676.543	22.533
Plant N	942.679	11.104
Plant P	0.030	0.899
Plant K	3996.641	20.104
Pn	4.025	14.163
Tr	0.040	5.330

Ci	0.476	0.322
GS	0	5.644
SPAD	2.442	6.818
Chlorophyll a	0.010	4.535
Chlorophyll b	0.001	3.168
Carotenoids	0.001	9.643
Total Chlorophyll	0.16	4.252
Shoot Cd concentration	0.003	5.858
Root Cd concentration	0.007	5.232
H_2O_2	11.055	8.868
MDA	0	4.316
TF	0.021	4.147
BCF	0.015	5.809
POD	34.466	1.527
SOD	35.483	0.673
CAT	44.892	9.596
GSH	30.259	8.822
Richness	9517.944	0.579
Shannon	0.001	0.164
Chao1	9501.241	0.579
